# Long-term changes of spine dynamics and microglia after transient peripheral immune response triggered by LPS *in vivo*

**DOI:** 10.1186/1756-6606-4-27

**Published:** 2011-06-17

**Authors:** Satoru Kondo, Shinichi Kohsaka, Shigeo Okabe

**Affiliations:** 1 Department of Cellular Neurobiology, Graduate School of Medicine, University of Tokyo, Bunkyo-ku, Tokyo 113-0033, Japan; 2 Department of Neurochemistry, National Institute of Neuroscience, Kodaira, Tokyo 187-8502, Japan

**Keywords:** Peripheral inflammation, Spine, Microglia, *in vivo *imaging, Sepsis

## Abstract

**Background:**

An episode of peripheral immune response may create long-lasting alterations in the neural network. Recent studies indicate a glial involvement in synaptic remodeling. Therefore it is postulated that both synaptic and glial changes could occur under the peripheral inflammation.

**Results:**

We tested this possibility by *in vivo *two-photon microscopy of dendritic spines after induction of a peripheral immune response by lipopolysaccharide (LPS) treatment of mice.

We observed that the spines were less stable in LPS-treated mice. The accumulation of spine changes gradually progressed and remained low over a week after LPS treatment but became significantly larger at four weeks. Over eight weeks after LPS treatment, the fraction of eliminated spines amounted to 20% of the initial population and this persistent destabilization resulted in a reduction of the total spine density.

We next evaluated glial activation by LPS administration. Activation of microglia was confirmed by a persistent increase of Iba1 immunoreactivity. Morphological changes in microglia were observed two days after LPS administration and were partially recovered within one week but sustained over a long time period.

**Conclusions:**

These results indicate long-lasting aggravating effects of a single transient peripheral immune response on both spines and microglia. The parallel persistent alterations of both spine turnover and the state of microglia *in vivo *suggest the presence of a pathological mechanism that sustains the enhanced remodeling of neural networks weeks after peripheral immune responses. This pathological mechanism may also underlie long-lasting cognitive dysfunctions after septic encephalopathy in human patients.

## Background

Sepsis is a serious medical condition caused by a severe immune response to infection. Recent studies have demonstrated that patients recovered from septic conditions can have long-term cognitive impairment, including memory deficits and attention disorders [1]. How impairment of brain functions persists long after recovery from sepsis still remains to be poorly understood. Peripheral injection of bacterial lipopolysaccharide (LPS), endotoxin from gram-negative bacteria, mimics the pathological state of sepsis. LPS interacts with Toll-like receptors on macrophages and initiates production of a variety of proinflammatory cytokines including tumor necrosis factor (TNF)- α, interleukin (IL)-1β, and IL-6 [2,3]. These cytokines cross the blood-brain barrier, activate cells of the brain capillaries, and also activate vagal afferents. Subsequently, these responses further induce the synthesis of prostaglandins [4-6] and cytokines within the brain that cause fever and general feelings of illness [7].

Consistent with cognitive impairment in human subjects who recovered from sepsis, peripheral administration of LPS in rodents has been shown to affect cognitive functions [8]. In line with these findings, LPS has also been demonstrated to impair long-term potentiation (LTP), a key cellular process in learning and memory [9,10]. How does LPS-evoked peripheral inflammation affect functions of the neural network? One obvious possibility is through alterations of glia [11]. Microglial cells are resident immune cells in the CNS [12,13] and have the ability to respond to inflammation by changing their shape from ramified to amoeboid morphology [14] or by changing the expression profile of marker molecules, such as ionized calcium binding adaptor molecule 1 (Iba1) [15,16]. Recent *in vivo *imaging studies reported rapid motility of microglial processes [17,18]. Direct contact of microglia with synapses may also be involved in synapse stripping in pathological conditions [19].

The neuronal network in the mammalian forebrain has been shown to be intrinsically dynamic [20,21] and *in vivo *time-lapse two-photon microscopy of dendritic spines in the mouse neocortex has provided direct evidence of network remodeling [22,23]. Although previous *in vivo *imaging studies reported remodeling of synapses after induction of ischemia [24,25] and in neurodegenerative conditions [26,27], long-lasting changes of synapses triggered by peripheral inflammatory responses have not yet been investigated. Here we performed *in vivo *spine imaging after LPS treatment. Surprisingly, mild and transient peripheral inflammation induced long-lasting changes of spine dynamics, associated with persistent up-regulation of Iba1 expression in microglia. The parallel sustained alterations of both spine turnover and the state of microglia *in vivo *may underlie long-term cognitive impairment after septic encephalopathy in human patients.

## Methods

### Animals

All experimental procedures were carried out in compliance with the institutional guidelines of the University of Tokyo and the government. This study was approved by the animal welfare ethics committee at the University of Tokyo, Faculty of Medicine with the approval ID of P08-016. Every effort was made to minimize the suffering and the number of animals used.

For *in vivo *imaging and histochemical analyses, both male and female C57BL/6 transgenic mice aged two to three months were used. For *in vivo *spine imaging, transgenic mice expressing green fluorescence protein (GFP) under the control of the *Thy1 *promoter (*Thy1-GFP *M mice) were used [28]. To visualize microglia, transgenic mice expressing GFP under the control of the *Iba1 *promoter (*Iba1-GFP *mice) were used [29].

### Drug treatment

*E.coli *lipopolysaccharide (LPS, strain O111:B4, Sigma-Aldrich (catalog number L4391)) was dissolved in a saline solution at a concentration of 0.2 mg/mL and stored at -30°C in small aliquots. Mice were intraperitoneally injected with a single dose of LPS (0.5 mg/kg). The optimal dose of LPS was determined from the morphological changes in microglia two days after LPS injection with doses of 0.1, 0.3, 0.5 or 5.0 mg/kg. We observed morphological changes in microglia at doses higher than 0.5 mg/kg. Injection of LPS at 5.0 mg/kg induced severe behavioral responses, while sickness behavior was less prominent at lower doses. From these pilot experiments, we selected a dose of 0.5 mg/kg for experiments with spine imaging and glial activation.

### Surgery

Mice were deeply anesthetized intraperitoneally with ketamine (100 mg/kg body weight) and xylazine (10 mg/kg body weight) diluted in a saline solution. After the disappearance of the pinching response the hair of the scalp was shaved and a midline incision of the scalp was made. Periosteum tissue was removed with a surgical blade and the somatosensory area (-1.5 mm from Bregma and 2.0 mm from the midline) was marked by stereotactic coordinates. A small rectangular metal plate with a round hole was glued on the skull and mice were fixed to the immobilized stage (SR-5M, Narishige) with a heating pad to maintain body temperature. The skull above the imaging area was thinned through the round hole of the metal plate. The thinning was initially done over a small area (a 1.5 × 1.5 mm square) with a high speed micro-drill (KM11, Minimo). When the bone reached ~50 μm in thickness, further thinning was performed manually with micro surgical blades (NORDLAND blade, Salvin Dental) until the skull reached ~15 μm in thickness. We paid particular attention not to push the skull during the thinning process. The final imaging window was a 0.5 × 0.5 mm square. For the repetitive imaging, the brain vasculature pattern was recorded with a CCD camera (GZ-MG70, Victor).

### *In vivo *imaging

A scanning microscope (FV-300, Olympus) equipped with a pulsed laser (MaiTai HP, Spectra Physics) was used for imaging with a water immersion objective lens (1.05 NA, 25x, Olympus). The wavelength was 920 nm and the average power of the laser after the objective lens was between 10 and 20 mW. The imaging area was 234 μm × 234 μm (low magnification) or 78 μm × 78 μm (high magnification), with an imaging depth 50 μm from the surface of the neocortex (Layer 1) and the step size of the z stack set to 0.75 μm. The pixel sizes of single horizontal images were set to 512 × 512. Low magnification images, together with images of the vasculature pattern taken with a CCD camera, served as the reference maps for repetitive acquisition of higher magnification images from the same cortical area. For repeated imaging, the metal plate attached on the skull was removed and the skin was sutured. The mice were kept on the heating pad until they recovered from the anesthesia and were returned to their home cage. To minimize the damage to the brain tissue due to the re-thinning of the skull, mice were imaged twice for most of the experiments and three times at the maximum.

### Fixation of animals and immunohistochemistry

Wild type or *Iba1-GFP *mice were sampled before or two, seven, 28 days after LPS injection. Mice were deeply anesthetized with pentobarbital and perfused transcardially with PBS followed by 4% paraformaldehyde. Brains were removed and further fixed in 4% paraformaldehyde overnight at 4°C. Slices were made with a vibratome (DTK-1000, Dosaka EM) with a 50 μm thickness. Slices from wild type mice were stained with anti-Iba1 antibody (1/500, Wako Pure Chemicals) or anti-glial fibrillary acidic protein (GFAP) antibody (1/3000, Sigma-Aldrich) followed by the fluorescence conjugated secondary antibodies to visualize the microglia or astrocytes. Mice from various time intervals after LPS injection or control were fixed on the same day and slices were stained at the same time. Images were obtained by using a laser scanning confocal microscope (FV-1000, Olympus) or a wide-field fluorescence microscope (BX-50, Olympus) under the same illumination and collection conditions.

### Data analysis

**Figure 1:**
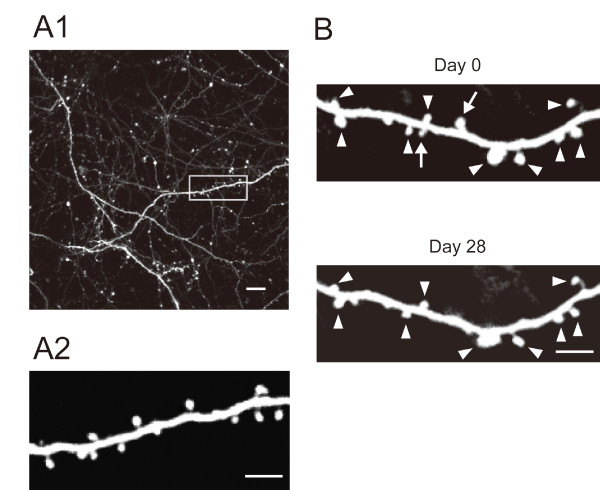
**Two-photon microscopy of dendritic spines**. (A1) Typical morphologies of dendrites in the superficial layer of the neocortex detected by *in vivo *two-photon microscopy. This is a projection image of z-stacks. Scale bar, 10 μm. (A2) Higher magnification view of the dendritic segment marked by the rectangle in A1. The image was generated from a montage of several adjacent optical sections separated by 0.75 μm in the Z-direction. Scale bar, 5 μm. (B) *In vivo *imaging of dendritic segments at two time points (day 0 and day 28). These images were also generated from a montage of several adjacent optical sections separated by 0.75 μm in z-direction, illustrating stable spines (arrowheads) and eliminated spines (arrows). Scale bar, 5 μm.

All analyses of spine dynamics, densities, and estimations of sizes were done manually using National Institutes of Health ImageJ software (http://rsb.info.nih.gov/ij). We could identify both spines and filopodia [30] but analyzed only dendritic protrusions classified as spines in this study (Figure 1) [31]. The same dendritic segments (5-50 μm length) were identified from the image stacks at different time points and spines were selected and classified into three groups. New spines were those identified only at the second time point. Eliminated spines were those present only at the first time point but missing at later points. Spines present at both time points were categorized as stable spines. The number of spines in each group was counted and both the formation and elimination rates were calculated as the percentages of eliminated spines and newly formed spines to the total number of spines examined respectively. To ensure the tissue movements and rotations three-dimensional stacks were always used for the analysis. We did not analyze structures that projected mainly along the imaging axis, below or above dendrites. We considered a spine in the second image to be the same spine as in the first image if the second image spine was located within 0.5 μm of the expected location based on its spatial relationship to adjacent spines or landmarks like axonal and dendritic orientation.

For the morphological analysis of spines, we selected dendritic segments where eliminated spines could be identified. Binary images were constructed by appropriate thresholding to determine the outline of spines. The length from the neck to the tip of a spine (a) and the maximum width of a head (b) were measured manually and the sum of these two values was taken as a parameter reflecting spine size.

For the spine density analysis, we used the same dendritic segments as in the spine turnover analysis. We selected the dendritic segments whose lengths were more than 20 μm for the spine density calculation. Spine density was calculated as (N of spines)/(lengths of dendritic segments).

For the analysis of Iba1 expression we took 41 images at the z step of 1 μm and a maximum projection image was generated from the same number of stack images for all the individual slices. Images were obtained with a laser scanning confocal microscope (FV-1000, Olympus) under the same illumination and collection conditions. The total fluorescence intensity of projection image was measured by ImageJ software. The background intensity was subtracted from the total fluorescence intensity. The intensity values were normalized to the mean intensity value of the control.

The density of microglia was calculated from the same slices as used for the measurement of intensity. The number of Iba1-positive cell bodies was manually counted three-dimensionally from 41 image stacks. The number of microglia was normalized to the mean number of microglia in the control.

Imaris v6.1.3 (Bitplane, Zurich, Switzerland) was used for the three-dimensional analysis of microglial processes. Surface rendering of GFP-positive microglia from z-stacks of confocal microscope images was performed and the lengths of processes were determined by measuring the distance from the centroid of a cell body to the tip along the processes.

Structural dynamics of microglia were quantified from time-lapse imaging of soma and protrusions *in vivo*. Images were obtained every three min for 30 min. We took 51 images at the z step of one μm for each time-interval image. The analysis was performed on maximum-intensity projections of fluorescence image stacks. The same number of stack images was used for each cell analysis. The dynamics of microglia were evaluated by measuring the length of extension and retraction of microglial protrusions. Ten processes of microglia were randomly selected and the length of extension and retraction was measured to compare the overlay of two images at two different time points.

**Figure 2:**
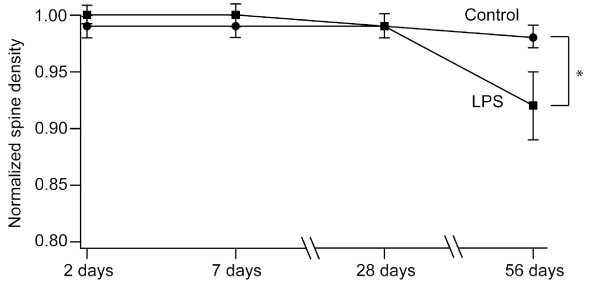
**Temporal change of spine density after LPS treatment**. Average spine densities at different time points after LPS treatment were calculated and normalized against the value at day zero (before LPS administration in the LPS-treated group). Spine densities were maintained close to the original value until 28 days after LPS injection. However, there was a late decline of spine density in LPS-treated group and the difference was statistically significant (*, p < 0.05).

**Figure 3:**
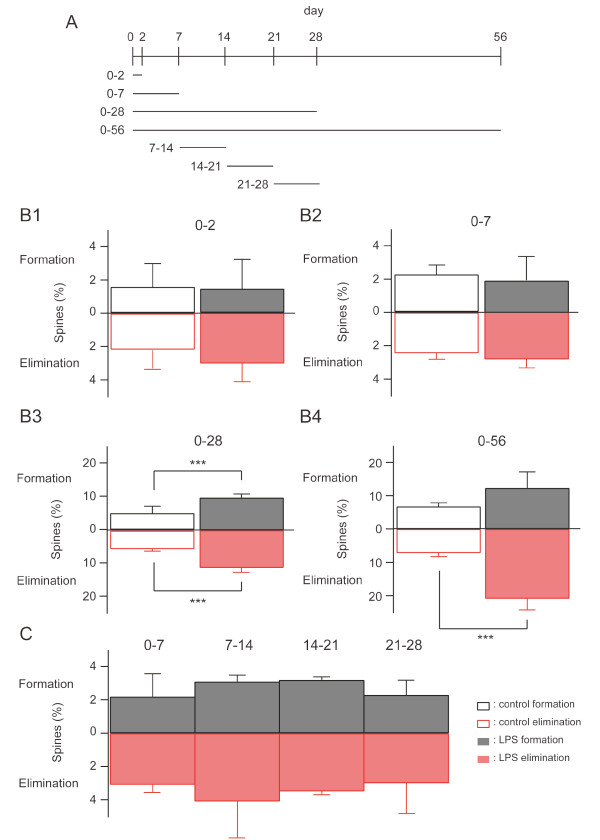
**Persistent up-regulation of spine turnover in mice treated with LPS**. (A) Spine turnover rates were measured as illustrated in the scheme. (B) Spine turnover rates of control and LPS-treated mice measured at two, seven, 28, and 56 days. Upward bars (black) and downward bars (red) represent the formation and elimination rate of spines, respectively. Spines formed and eliminated in control: 2.4 ± 1.4% and 2.5 ± 1.1% over two days (N = 6) (B1), 2.6 ± 0.4% and 2.7 ± 0.6% over seven days (N = 6) (B2), 5.2 ± 2.1% and 5.5 ± 0.6% over 28 days (N = 6) (B3), 6.8 ± 1.1% and 7.2 ± 1.1% over 56 days (N = 3) (B4). Spines formed and eliminated after LPS injection: 1.7 ± 1.8% and 3.3 ± 1.1% over two days (N = 3) (B1), 2.2 ± 1.4% and 3.1 ± 0.5% over seven days (N = 3) (B2), 10.7 ± 1.5% and 11.0 ± 1.2% over 28 days (N = 6) (B3), 12.4 ± 4.9% and 20.9 ± 3.5% over 56 days (N = 4) (B4). These data were statistically compared between control and LPS treatment (spines formed: p > 0.5 for two days; p > 0.5 for seven days; p < 0.001 for 28 days; p > 0.1 for 56 days; spines eliminated: p > 0.1 for two days; p > 0.1 for seven days; p < 0.001 for 28 days; p < 0.001 for 56 days). ***, p < 0.001. (C) Spine turnover measured with a fixed interval but with different days of the first imaging.

**Figure 4:**
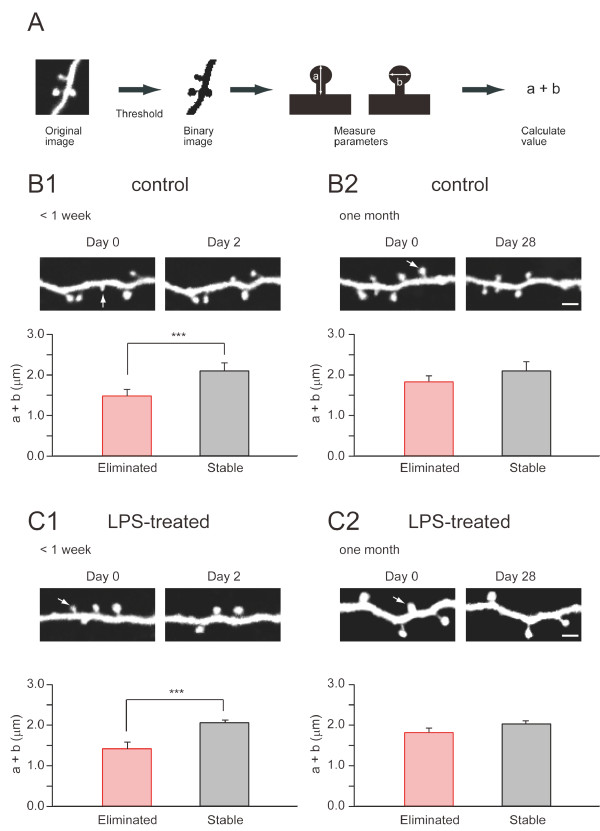
**Relationship between spine size and spine elimination**. (A) The sizes (both length (a) and width (b)) of spines were measured as illustrated. The sum of the values "a" and "b" was calculated and utilized as an index of spine size. (B1) Spines eliminated within two or seven days were smaller than the spines maintained for the same period. Images are comparisons over two days. An arrow indicates an eliminated spine. The bar graph shows the combined data from images taken over two or seven days, as the frequency distribution of each population was indistinguishable. (B2) When spines were imaged with intervals of 28 days, the sizes of eliminated spines were similar to those of stable spines. An arrow indicates an eliminated spine. (C1) Spines eliminated within two or seven days were smaller than the stable spines also in the LPS-treated group. An arrow indicates an eliminated spine. (C2) Spines eliminated 28 days after LPS treatment had comparable sizes with those maintained. An arrow indicates an eliminated spine. Importantly, the average sizes of eliminated spines between control and LPS-treated groups were not different at two different time points (less than seven days in B1/C1, at 28 days in B2/C2), indicating that the effect of LPS is not selective for a specific size of spines. ***, p < 0.001. Scale bars, 2 μm.

**Figure 5:**
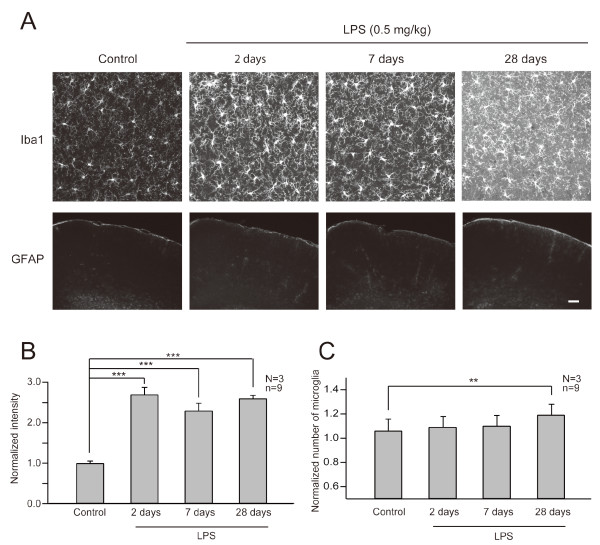
**LPS induced persistent activation of microglia**. (A) Immunohistochemical analyses were performed with fixed cortical slices from control and LPS treated mice at different time points. Slices were stained either with anti-Iba1 antibody for microglia or anti-GFAP antibody for astrocytes. Projection images are shown. Scale bar in the upper column, 10 μm; bar in the lower column, 400 μm. (B) Temporal profile of enhanced Iba1 expression after LPS treatment. To evaluate expression of Iba1, the total intensity of fluorescence was measured and the averages were plotted as values normalized to the control (N: number of mice, n: number of slices). The intensity of fluorescence was significantly increased after two days of LPS treatment and this increasing trend was maintained until 28 days. (C) Late increase in the number of microglia after LPS treatment. The number of microglia was counted and the averages were plotted as values normalized to the control (N: number of mice, n: number of slices). The number of microglia was significantly increased only at 28 days after LPS treatment. **, p < 0.01, ***, p < 0.001.

**Figure 6:**
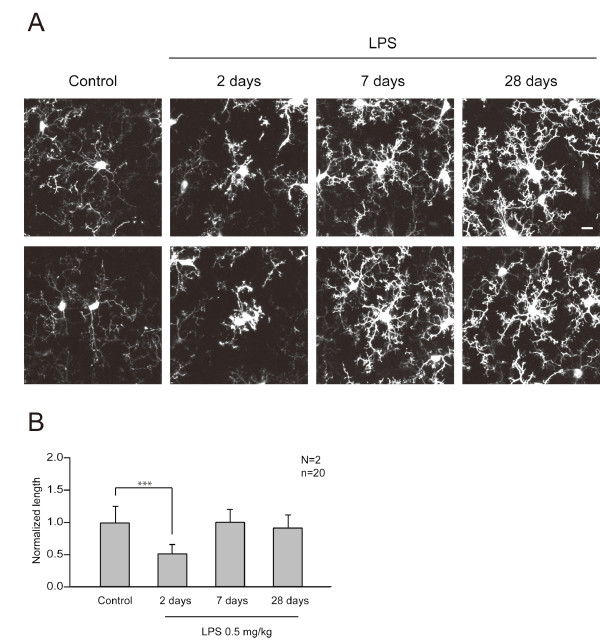
**Morphological changes of microglia after LPS treatment**. (A) High magnification images of microglia in *Iba1-GFP *mice. Mice were either unperturbed or received LPS injection and were sacrificed for immunohistochemical analyses two, seven, and 28 days later. Scale bar, 10 μm. (B) Total lengths of microglial processes were measured and the averages are shown as lengths normalized to the control (N: number of mice, n: number of microglia). The shrinkage of processes was observed at two days but they recovered their original lengths seven days after LPS injection. GFP signals from the microglial processes were enhanced even at later time points (seven and 28 days), but this may be due to either real increase in process diameters or increases in the GFP protein content. ***, p < 0.001.

**Figure 7:**
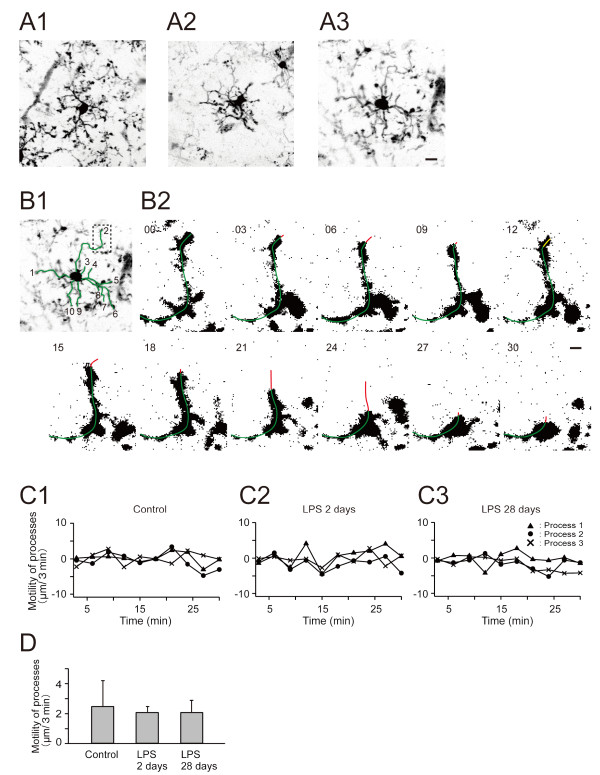
**Unaltered dynamics of microglial processes in LPS-treated mice**. (A) Morphology of microglia imaged by *in vivo *two-photon microscopy of *Iba1-GFP *mice. A1 shows a cell from a mouse without LPS administration. A2 and A3 show cells from LPS-treated mice at two and 28 days after LPS treatment, respectively. Scale bar, 10 μm. (B1) An example of a microglial cell utilized for time-lapse analysis (identical cell shown in A3). This image shows the morphology at zero time point, with an overlay of green lines (numbered from 1 to 10) on individual processes. The tips of these green lines were recorded and their distances between adjacent time frames (3 min intervals) were calculated as an index of process motility shown in C and D. (B2) Binary images of a microglial process (green line) extracted from time-lapse sequences in the image area marked by a dotted rectangle in B1, illustrating the extent (yellow line) of rapid growth and shrinkage (red line). Numbers in the upper left corner indicate elapsed time in min. Scale bar, 2 μm. (C) The motility of microglial processes. Extension and retraction of processes were plotted for three representative processes with time intervals of three min. Positive values indicate process extension and negative values indicate retraction. (D) Summation of absolute distances of extension and retraction during 30 minutes was calculated and the average speeds per frame (three min) were estimated from 10 processes of control and LPS-treated mice. The average motility was comparable among control and LPS-treated groups.

All the data are presented as means ± S.D. Multiple comparisons were made by an ANOVA test, followed by a Tukey's test (Figures 2, 3, 5, 6, 7). Statistical significance was evaluated using Student's t-test (Figure 4). Differences were considered to be significant if p < 0.05.

### Cytokine array analysis

Tissue extracts from control and LPS-treated mice were screened by using a cytokine antibody array kit (Array1, RayBiotech Inc., Atlanta, GA, USA) according to the manufacturer's instructions. After euthanasia of mice with a large dose of pentobarbital, the neocortex and the hippocampus were dissected from the forebrain and homogenized in a lysis buffer. Brain extracts were then centrifuged at 10,000 × g for 10 min at 4°C. Protein concentrations of supernatants were determined and equal amounts of protein were incubated with array membranes. The signals generated by enhanced chemiluminescence were recorded by ChemiDoc XRS (Bio-Rad, Tokyo, Japan). For the analysis of chemiluminescence signals from cytokine spots, total signals were calculated and then background intensity was subtracted. The averaged optical densities of the six positive controls on the membrane were calculated and the optical densities of cytokine spots were divided by these values. These normalized values were compared and the ratio between control and LPS-treated mice was calculated.

## Results

### Transient peripheral immune response induces long-lasting changes of spine dynamics *in vivo*

Turnover of excitatory synapses in the neocortex with or without stimulation of the peripheral immune system can be quantitatively evaluated by two-photon imaging of dendritic spines *in vivo*. We visualized apical dendrites of pyramidal neurons in the somatosensory cortex of adult transgenic mice expressing GFP under the control of the *Thy1 *promoter in a small subset of the CNS neurons (*Thy1-GFP *M mice) [28]. We selected the thinned-skull method in this study, because of the less prominent effects of this surgical procedure on the activation of glial cells [32]. To detect spines generated and eliminated, we obtained image stacks of GFP-filled dendrites by two-photon microscopy at two time points separated by two days, seven days, four weeks or eight weeks (Figure 1A). A pair of image stacks was compared and spines present in both image stacks, and those present only at a single time point were identified manually (Figure 1B). After identification and classification of spines, the fractions of newly formed spines and eliminated spines were calculated. The fraction of spines added or eliminated in unperturbed animals was low and comparable to a previous report [33]. The fraction of dynamic spines was less than 5% over a period of seven days, 5% over four weeks, and still less than 10% over a period of eight weeks (see Figure 3B).

We challenged *Thy1-GFP *M mice with peripheral administration of LPS and measured the turnover rate of spines. The dose of LPS (0.5 mg/kg) was set to the minimal dose sufficient for up-regulation of Iba-1, which is a well-characterized marker of microglial activation. The protocols for imaging were set to be identical for control and LPS-treated groups, except that the LPS-treated group received a single intraperitoneal injection of LPS after acquisition of spine images on the first day. We first determined if there were any alterations in spine densities after LPS administration (Figure 2). Spine densities (estimated from images taken over intervals from two days to four weeks within the same volume of the cortex) did not show prominent differences, with a tendency for a slight decline [33,34]. However, there was a significant reduction in spine density at eight weeks after LPS treatment, indicating a late impact of LPS treatment on the cortical network.

Delayed changes in the total spine density may be derived from persistent changes in the spine addition/elimination rates triggered by LPS. To test this possibility, we calculated the fractions of added or eliminated spines (Figure 3A). Within the first week after LPS treatment, these dynamic fractions were not significantly larger than the corresponding fractions in the control group (Figure 3B). However, when we waited for four weeks, the fractions of added or eliminated spines accumulated after LPS treatment were significantly larger than the corresponding fractions in the control group (5.5% (control) versus 11.0% (LPS-treated) for spine elimination, 5.2% (control) versus 10.7% (LPS-treated) for spine formation at four weeks). Spine elimination was further enhanced by LPS treatment when we waited for eight weeks (7.2% (control) versus 20.9% (LPS-treated)), but the extent of spine addition did not match that of elimination in the LPS-treated group (6.8% (control) versus 12.4% (LPS-treated)). This unbalance of addition and elimination of spines could explain the reduction of the total spine density at eight weeks after LPS treatment.

The selective increase of the dynamic spine fraction at late time points could be explained by either an accumulation of small changes gradually over time or by abrupt up-regulation of dynamics at specific time points. To discriminate between these two possibilities, we imaged dendritic spines with intervals fixed to one week, but altered the first imaging days to be either zero, seven, 14, or 21 days after LPS treatment. We could not detect up-regulation of spine dynamics in any of these time segments (Figure 3C), supporting the first possibility that the accumulation of small changes only becomes obvious four weeks after LPS treatment. In other words, these results indicate the presence of persistent alterations in spine dynamics lasting well beyond several weeks after transient and mild activation of the peripheral immune system.

### Morphological characteristics of eliminated spines

Next, we evaluated morphological characteristics of spines eliminated early (within one week) or late (later than four weeks) after the initial time point of the spine imaging. Previous reports on spine lifetime in slices indicated that spines with larger volumes had longer lifetimes [35]. Therefore, small spines may have a higher probability for elimination after LPS treatment *in vivo*. On the other hand, if spine elimination is a stochastic process with no preference for size, we might not detect any differences in spine size between groups with different lifetimes. To discriminate between these possibilities, we evaluated the morphology of individual spines by calculating the sum of their lengths and widths (Figure 4A). This measurement suggests that indeed there exists differences in morphology between spines eliminated within one week and those that remained (Figure 4B1). Eliminated spines tended to be smaller than the stable ones (p < 0.001). However, a similar comparison between spines eliminated within one month and those that survived for one month did not show such a tendency (Figure 4B2). Thus, we consider the spines with a life-time of less than a week morphologically distinct from the remaining spine population.

LPS treatment increased the fraction of added and eliminated spines only in a late phase. At four weeks after LPS treatment, the fraction of eliminated spines was 10%, twice as large as the fraction in the control (5%). If LPS treatment affected a subset of spines, such as those with smaller volumes, we might be able to detect changes in the frequency distribution of spines eliminated late after LPS treatment. However, the morphology of spines eliminated within one week or one month was not significantly different between the LPS-treated group and the control group (Figure 4B and 4C), suggesting that there was no preferential impact of LPS treatment on a subset of spines with a specific morphology.

We conclude that spines destined to be eliminated within a week are smaller than the average in both control and LPS-treated conditions. LPS increased the fraction of spines to be eliminated late after LPS treatment, but the morphological difference of this LPS-sensitive fraction could not be detected by comparison of spine images, indicating that the late effect of LPS is to increase the probability of spine elimination irrespective of the spine size.

### LPS-induced alterations in glial properties

The *in vivo *imaging of LPS-treated *Thy1-GFP *M mice indicated the presence of persistent alterations in spine dynamics lasting well beyond several weeks after transient activation of the peripheral immune system. Peripheral immune responses can secondarily induce changes in glial components in the CNS, especially in astrocytes and microglial cells [36,37]. We performed immunohistochemistry of the neocortex two, seven and 28 days after LPS treatment to detect activation of astrocytes and microglial cells. We could not detect up-regulation of the astrocytic marker GFAP. However, Iba1, a marker of microglial activation, was up-regulated at all time points examined (p < 0.001 for two, seven, 28 days) (Figure 5A and 5B). Notably, up-regulated Iba1 immunoreactivity was maintained even at 28 days after LPS injection. There was also a specific increase of microglial density at 28 days after LPS treatment (p < 0.01) (Figure 5C). These results indicate prolonged activation and late proliferation of microglia following a single peripheral administration of LPS.

Peripheral injection of LPS may induce morphological changes of microglia. To test this possibility, we measured the length of microglial processes using a transgenic mouse line expressing GFP in microglia under the control of the *Iba1 *enhancer element (*Iba1-GFP *mice) [29]. Cytoplasmic GFP signals from microglia in *Iba1-GFP *mice reflect cellular morphology more precisely than the immunoreactivity of endogenous Iba1, which may reflect both cell shape and intracellular distribution of the protein. There was a transient shrinkage of microglial processes assessed by GFP signal two days after LPS treatment (p < 0.001), but the processes showed re-extension at seven and 28 days after LPS treatment (Figure 6). Consistent with the up-regulation of the endogenous *Iba1 *gene, GFP expression under the control of the *Iba1 *promoter also increased progressively after LPS treatment.

Microglia retracted their processes two days after LPS treatment but recovered from the initial shrinkage of their processes at time points later than seven days. A previous study reported the importance of process motility and contact with synapses for the regulation of synapse remodeling by microglia [19]. To evaluate the extent of microglial process motility, we visualized the dynamics of microglial processes using *in vivo *two-photon microscopy of *Iba1-GFP *mice. Time-lapse images were obtained every three min for 30 min. The temporal profiles of process extension and retraction were created from images of microglia without stimulation (Figure 7A1), microglia two days after LPS treatment (Figure 7A2), and 28 days after LPS treatment (Figure 7A3). The average velocities of process remodeling determined from time-lapse images (Figure 7B) were similar among groups with or without LPS treatment and groups with different intervals between LPS treatment and *in vivo *imaging (Figure 7C and 7D). These observations indicate microglia in both acute and chronic phases after LPS treatment are able to interact with synapses with their highly dynamic processes.

### Brain cytokine and chemokine profiles after LPS treatment

**Figure 8:**
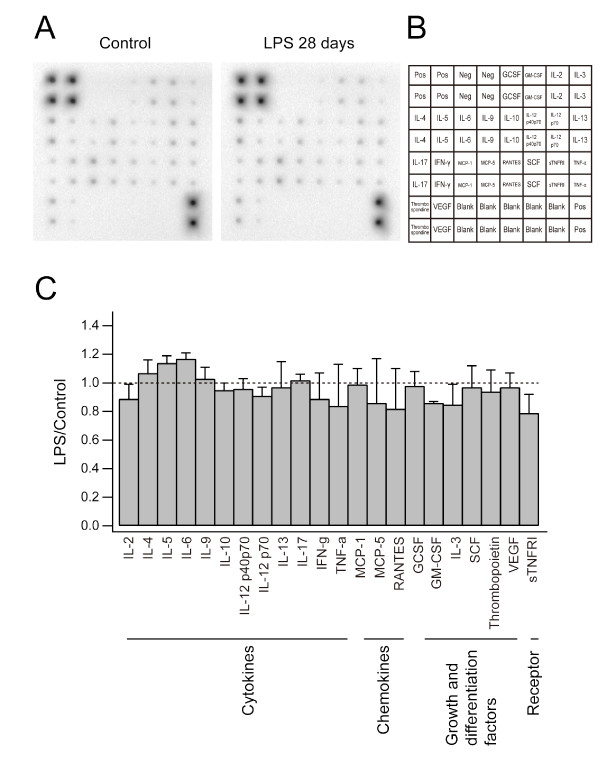
**Brain cytokine expression after LPS treatment**. (A) Images of cytokine array membranes incubated with brain homogenates from control mice (N = 3) and mice treated with LPS 28 days before preparation of brain homogenates (N = 3). (B) The arrangement of tested cytokines on the membrane. (C) The density of each spot was measured and the ratios between control and LPS-treated groups were calculated. There was no substantial increase or decrease in expression of the cytokines we tested.

We next asked whether soluble factors, such as TNF-α and IL-6, in the neocortex were persistently modulated after LPS treatment. Inflammation induces the expression of a variety of cytokines and chemokines in microglia [38]. Profiles of 22 different cytokines and chemokines in lysates taken from the neocortex of mice were evaluated by using antibody array membranes. We prepared brain lysate from mice sacrificed at multiple time points (one hr, two days, or 28 days) after LPS treatment (N = 1 for one hr and two days (data not shown), N = 3 for 28 days) and from control mice (N = 3). Signals were detected by a chemiluminescence method and were compared with the signal intensity of control mice (Figure 8A and 8B). We did not detect more than a two-fold increase or decrease in the level of cytokines/chemokines tested at any time point (Figure 8C). To test the sensitivity of our membrane array system, we challenged the mice with a ten-fold higher dose of LPS (5 mg/kg) (N = 1) that reportedly induces the amoeboid transformation of microglia and up-regulation of cytokines. In this condition, we successfully detected the up regulation of some cytokines (IL-6, IL-12, and TNF-α; data not shown) as reported previously [37]. This demonstrated that the detection system was indeed capable of measuring marked changes in cytokines/chemokines. Thus, at the LPS dose used in our imaging experiments, spine turnover rate might be modulated by other soluble factors secreted from microglia. However, we can not exclude the possibility that activated microglia, which corresponds to a small fraction of the cells present in the neocortex, secreted factors whose amount is under the limit of sensitivity of our detection system.

## Discussion

In this study, we addressed the question of how peripheral immune responses can modulate network remodeling of the neocortex *in vivo*. We imaged dendritic spines *in vivo *and analyzed spine dynamics over a period of several days to weeks after transient activation of the peripheral immune system with LPS. Within a week after LPS treatment, spine dynamics were low and comparable to those in control mice, indicating a minimal acute effect of systemic LPS treatment. Surprisingly, the fraction of newly formed or eliminated spines accumulated during four weeks after LPS treatment was twice as large as the proportion in control mice. The enhanced spine turnover was associated with persistent activation of microglial cells. The persistent modification of both spine dynamics and microglial activity suggests long-lasting effects of a single transient peripheral immune response on brain functions.

### Peripheral immune response triggers sustained up-regulation of spine turnover *in vivo*

*In vivo *imaging of dendritic spines can be achieved by two different surgical techniques. The first approach, using chronic open-skull glass windows, provides clear imaging windows for efficient two-photon excitation in the deeper cortical layers without a limit on imaging time points [31]. The second approach, using thinned skull windows, gives less efficient excitation of the deep cortical layers and is limited in the number of repetitive imaging, due to the technical difficulty of thinning the same cranial tissue multiple times [22]. Previous reports indicate that the open-skull window is associated with transient glial activation, which is not present after proper thinned-skull surgery [32]. Because our research goal was to assess the influence of mild peripheral immune response on the remodeling of the neural network, we had to exclude any possibility of inducing inflammation in the brain parenchyma due to the imaging protocol itself. Therefore, we imaged dendritic spines through a thinned-skull window in this study and successfully confirmed the remarkably stable nature of dendritic spines (turnover of < 10% over one month) [22].

In order to activate the peripheral immune system, we administered a single low dose of LPS intraperitoneally. This treatment induced persistent enhancement of spine turnover in vivo. We used the lowest possible dose of LPS (0.5 mg/kg) to induce up-regulation of Iba-1 immunoreactivity in microglia and there was no sign of acute neuronal degeneration in the brain (data not shown). A previous study by Sparkman *et al*. using a single intraperitoneal injection of LPS at the low dose of 0.25 mg/kg reported the presence of classic signs of sickness behavior, including decreased locomotion, hunched posture, piloerection, and anorexia [39]. Therefore we cannot eliminate the possibility that emotional and cognitive alterations acutely induced by sickness-related behavioral changes are partly responsible for alterations in spine dynamics. On later days of in vivo imaging, however, mice are fully recovered and it is less likely that the initial sickness behavior is the direct cause of the enduring enhancement of spine dynamics. Instead, we propose that persistent activation of microglia over a period of more than several weeks plays an important role in enhanced synapse remodeling.

Synapse formation and elimination are believed to be the basis for the plastic changes of neuronal circuits [40,41]. Therefore changes in synaptic connections should be associated with functional changes in the neural network. Then, what are the functional outcomes of spine dynamics after LPS treatment? Interestingly, Sparkman *et al*. reported that LPS administration affected the performance of mice in the Morris water maze more profoundly on later test days [39]. The difference in distance swam became significant only after three days had elapsed following LPS injection. This time course may reflect accumulation of network remodeling in the hippocampus over the period of several days, which may be paralleled by the enhanced spine turnover we observed in the neocortex. A possible link between cognitive impairment observed in LPS-treated rodents and alterations in the neural network is reinforced by a previous study showing LPS-dependent modification of synaptic plasticity, such as LTP [9]. For example, Shaw *et al*. reported impairment of both the dentate gyrus LTP *in vivo *and performance of a water maze task in rats that received a single dose of LPS (0.25 mg/kg) intraperitoneally [42]. Cytokines up-regulated by LPS administration, such as TNFα and IL-1β, are also known to function as modulators of synaptic plasticity [43-45]. It is possible that elevation of cytokines initiated by peripheral LPS treatment modulates LTP/long-term depression (LTD)-like mechanisms *in vivo *and subsequently triggers structural remodeling of the neural network, including addition and elimination of spines.

### Persistent microglial activation triggered by peripheral immune responses may be involved in enhanced spine dynamics

Neuronal networks in the mammalian forebrain are intrinsically dynamic [20,46] and are regulated by interactions with glia [19,47-49]. Microglial cells constitute the major cell type which responds to peripheral inflammation in the CNS [37]. Therefore we suspected that microglial cells directly modulated spine dynamics in response to intraperitoneal administration of LPS. As we already discussed, *in vivo *imaging through an open-skull glass window results in relatively high turnover rate of spines, together with transient activation of microglia. In contrast, with the thinned-skull window technique, much less spine dynamics and microglia activation were observed [32]. In pathological states, microglial processes show enhanced motility [17,18] and preferentially associate with synapses and may facilitate their deconstruction [19]. How microglia regulate neural networks *in vivo *is still poorly understood. Microglia may induce synapse remodeling via direct mechanical contact of their processes with synapses, or they may release soluble factors which destabilize nearby synapses. In order to evaluate these possibilities, we performed morphological and biochemical analyses of microglia in the neocortex after LPS administration.

In order to address the structural interactions between microglia and spines, we first analyzed the morphological transition of microglia with enhanced Iba1 expression after LPS administration. Iba1 is a small EF hand calcium binding protein involved in Rac and calcium signaling pathways [15]. Therefore, morphological changes of microglia could be related to the reorganization of the actin network through the Rac pathway. We observed both enhanced Iba1 expression and shrinkage of microglial processes two days after LPS treatment. This observation confirms that a single peripheral injection of LPS is sufficient to trigger activation of microglia. Iba1 expression was up-regulated between seven and 28 days after LPS administration and the number of microglia was also increased during the same period. These observations are consistent with the idea that microglia activation is not only maintained, but gradually enhanced over a time period of several weeks after LPS treatment. Our *in vivo *imaging of *Iba1-GFP *mice revealed that microglial processes had comparable motility before and after LPS treatment. Because the density of microglia increased late after LPS treatment, the frequency of individual synapses to receive contact of activated microglia may also be enhanced. This effect possibly underlies the progressive elimination of spines after LPS treatment. In the current studies, we restricted our analysis of microglial changes up to one month and we did not investigate the direct association between dendritic spines and microglial processes. However, the dynamic nature of microglial processes and their interaction with synapses were reported recently (17-19). Our present observation of the alterations in spine remodeling suggests an association with the change in microglia. Further studies are necessary to elucidate whether microglial contact is involved in the spine elimination.

Cytokines that are secreted from glial cells in the brain can affect both synaptic functions and spine morphology [43,44,50]. In animal models of neurodegenerative disorders and aging, there are reports of proliferating activated microglia and the increased levels of proinflammatory and anti-inflammatory cytokines together with impairments of spine density and morphology [51]. Systemic administration of LPS also increases several types of cytokines in the peripheral blood, mainly TNF-α, IL-1β, and IL-6 [7]. In order to detect soluble factors up-regulated in the brain during the persistent enhancement of spine turnover, we measured the levels of cytokines and chemokines in brain extracts prepared 28 days after LPS treatment. However, we were unable to detect increased expression of multiple cytokines. These results suggest that either signals mediated by soluble factors may not be involved in synapse destabilization or, alternatively, factors we have not yet tested play a critical role. It is also possible that cytokines or chemokines we tested are indeed involved in synapse remodeling, but the concentration sufficient for exerting their effect is below the limit of detection.

In this study, we found that mild and transient peripheral inflammation induced long-lasting changes of spine dynamics over a period of several weeks and persistent up-regulation of Iba1 expression in microglia. Although we could not demonstrate convincing evidence for the interrelationship between these two alterations, recent findings showing the interaction between microglia and synapses (17-19) indicate that activation of microglia may be associated with spine remodeling. However, the relationship between microglia and spine changes in our present study remains to be fully elucidated in future experiments. The parallel sustained alterations of both spine turnover and the state of microglia in vivo may underlie long-term cognitive impairment after peripheral inflammation, which may be relevant to the neurological problems of patients who have recovered from septic conditions.

## Conclusions

In this study, we used *in vivo *two-photon imaging in mice to test whether peripheral immune responses affected cortical synapses. We observed parallel increases in spine dynamics and microglial activities after LPS treatment. Although a more definitive causal relationship between spine and microglial changes should be clarified in the future, our results implicate that microglial activities may affect spine dynamics. These observations could help our understanding of the long-term cognitive impairment of septic human patients.

## List of abbreviations used

GFAP: glial fibrillary acidic protein; GFP: green fluorescence protein; Iba: ionized calcium binding adaptor molecule; IL: interleukin; LPS: lipopolysaccharide; LTD: long-term depression; LTP: long-term potentiation; TNF: tumor necrosis factor.

## Competing interests

The authors declare that they have no competing interests.

## Authors' contributions

SatK conducted experiments, analyzed data, and wrote the manuscript. ShiK generated the *Iba1-GFP *transgenic mouse. SO designed experiments, and wrote the manuscript. All authors read and approved the final manuscript.
